# Particle Filtering-Based In-Flight Icing Detection for Unmanned Aerial Vehicles

**DOI:** 10.3390/s26061993

**Published:** 2026-03-23

**Authors:** Toufik Souanef, Mohamed Tadjine, Nadjim Horri, Ilyes Chaabeni, Bilel Boulassel

**Affiliations:** 1 Centre for Aeronautics, Cranfield University, College Road, Cranfield MK43 0AL, UK; toufik.souanef@cranfield.ac.uk; 2 Department of Electrical Engineering, Ecole Nationale Polytechnique (ENP), El Harrach, Algiers 16200, Algeria; mohamed.tadjine@g.enp.edu.dz (M.T.); ilyes.chaabeni@g.enp.edu.dz (I.C.); bilel.boulassel@g.enp.edu.dz (B.B.); 3 School of Engineering, University of Leicester, University Road, Leicester LE1 7RH, UK

**Keywords:** icing detection, fixed-wing UAV, particle filter, EKF, aerodynamic parameter estimation

## Abstract

Ice accretion poses a threat to fixed-wing aerial vehicles as it alters the wings’ shape and thus degrades the aerodynamic performance. In manned aircraft, the icing detection system assists the pilot and utilises dedicated sensors. However, in unmanned aerial vehicles (UAVs), onboard icing detection can generally only be achieved using standard sensors in conjunction with dynamical models, because dedicated sensors are rarely available. In this paper, we propose two approaches based on the particle filter for both icing detection and accurate state and aerodynamic parameter estimation in the presence of icing, with different levels of severity. The first approach uses the observation likelihood for icing hypothesis testing with a complement of the Gaussian kernel to compute icing probability. The second approach uses a discrete jump approach based on a Bernoulli process and a subset of particles to test the icing hypothesis for faster icing detection by estimating changes in icing-related aerodynamic parameters. Using both approaches, the simulation results demonstrate improved estimation accuracy compared to an extended Kalman filter (EKF), under both moderate and severe icing conditions. With adequate tuning, the proposed approaches show potential for indirect icing detection in UAVs. They also enable the computation of icing severity and provide a more accurate and reliable estimate of the icing probability compared to the EKF.

## 1. Introduction

In-flight icing accretion is a constant threat to aviation, as it can lead to fatal accidents in both manned and unmanned aircraft [1]. Icing has been studied for decades and is still not a very well-known phenomenon due to its stochastic nature and the difficulty of modelling, which often relies on expensive wind tunnel tests and computational fluid dynamics (CFD) simulations [2].

According to its shape, density, and environment, icing can be categorised into three main types: rime, glaze, and mixed icing. Rime ice forms at low freezing temperatures with low liquid water content, while glaze ice occurs at higher freezing temperatures with high water content, often creating dense, horn-like shapes that severely affect aerodynamic performance.

When icing forms on the wing surfaces of aircraft, typically the leading edge of the airfoil, its shape is altered, disrupting airflow. As a consequence, aerodynamic performance is degraded: drag increases, lift decreases, and oscillations around the longitudinal axis may occur due to a change in the pitching moment. In the worst case, icing can lead to stalling and cause the aircraft to crash. Icing can also degrade propeller performance by causing vibrations.

Although more work has been done for the case of manned aircraft, icing is more severe in the case of unmanned aerial vehicles (UAVs) due to their low speed, small size, and cost limit. In-flight icing detection methods for UAVs range from classical approaches like residual-based fault detection and isolation (FDI), estimation and optimal filters, statistical decision theory, to more modern approaches like artificial neural networks and classification-based FDI [3].

Methods based on aerodynamic parameter estimation require excitation of the UAV due to observability issues, such as altitude change [4], which may not always be permitted, and does not guarantee enough precision. Furthermore, their estimation may not be required to account for the power needed; once the de-icing system eliminates icing, the parameters will be back to their nominal values. Residual-based approaches require accurate state estimation, which may not be the case for UAVs when icing occurs. In addition, many methods are constrained by noise type (such as Gaussian noise) or linear approximation.

Many methods used for icing detection are based on nonlinear or linear observer assumptions that are corrupted by noise, such as using a bank of linear parameter-varying unknown input observers, as in [5], and super-twisting observers [6], which are not always realistic for capturing the nonlinearity and stochasticity of small UAV dynamics under icing conditions, especially at low speeds.

Other methods use system identification, as in [7], where an adaptive multiple model approach was used. Stochastic methods using filters other than the extended Kalman filter (EKF), which has been extensively used, are less common. In [8], an FDI scheme for propeller fault, including icing fault, was proposed, using a bank of EKFs for generating residuals and a bank of Bayes filters for analysis. Statistical decision theory has been applied only in the work of [9], in which the generalised likelihood ratio test was used based on accelerations and pitch rate residuals. A normal distribution was considered, and the MLE (maximum likelihood estimator) was used to estimate its parameters in both the null hypothesis and alternative hypothesis.

However, most of the research that has been done for icing detection for UAVs focuses on detection only, and does not evaluate the quality of the state estimates in the presence of icing. Additionally, state estimates are assumed to be known or estimated in some works, but their accuracy may not be guaranteed when icing is present. This is because when icing occurs, the change in the aerodynamic parameters can be large (up to 50% increase/decrease) [9], which causes the UAV model to be no longer accurate with the nominal parameters, and thus, the model-based estimators may lose track, leading to invalid state estimates. The proposed approaches combine icing detection with aerodynamic parameter estimation and icing severity evaluation.

The particle filter (PF) is particularly advantageous for UAV icing detection because it naturally handles the nonlinear, non-Gaussian dynamics introduced by icing accretion, unlike traditional methods that require linearisation and Gaussian noise assumptions. Furthermore, its ability to maintain multiple hypotheses simultaneously through particles enables robust state estimation even during large aerodynamic parameter shifts (up to 50%) where model-based estimators typically fail [9]. A jump-Markov regularised PF was also found to be efficient for scenarios, with the ability to jump between nominal and faulty sensor and actuator states and to estimate the faults [10]. A simpler discrete jump approach based on a Bernoulli process model is adopted in this paper, specifically for icing detection.

The proposed estimation approach employs a PF that explicitly incorporates aerodynamic degradation into the state space, enabling accurate state estimation and indirect ice detection. The estimation of icing-related aerodynamic degradation was also used in [6], where a super-twisting observer was designed under hypotheses applicable to civil aircraft. However, those assumptions cannot be applied to small unmanned UAVs. The novelty of our approach lies in using a probabilistic particle filtering approach to icing detection instead of a deterministic one, which allows the computation of the icing probability and its severity level, with application to the nonlinear dynamics and non-Gaussian models that characterise aircraft icing conditions [11].

Two particle filter-based icing detection approaches are proposed, with distinct detection and hypothesis testing methods. The first employs a Gaussian kernel to compute icing probability from aerodynamic deviations, with sensitivity controlled by tuned standard deviation parameters. The second utilises statistical decision theory, where a particle subset is used to test the change hypothesis with guided noise generation. A discrete jump indicator is incorporated into the state vector, and Gamma-distributed noise is generated when a jump is hypothesised. A similarity with [10] is the use of a small subset of particles to test the alternative (jump) hypothesis to the current one (icing present or not).

This indirect icing detection system is designed to minimise false alarms and missed detections, detect the icing shortly after its onset. These capabilities can contribute to UAV safety and integrity.

The main contributions of this work are as follows:

- This is the first application of particle filtering for UAV icing detection, handling nonlinear/non-Gaussian systems without linearisation.
- A dual icing detection framework is proposed based on Gaussian kernel probability and statistical decision theory with discrete jumps. When combined with particle filtering, the latter assigns a small probability (small subset of particles) to jumping to the alternative hypothesis using a gamma function-based noise model for abrupt aerodynamic parameter changes.
- Simultaneous detection and estimation is achieved, maintaining state accuracy during icing where traditional estimators fail.
- Validation is performed of the ability of the proposed particle filtering algorithms to accurately estimate aerodynamic parameters and to evaluate the icing probability under moderate to severe icing scenarios using standard UAV sensors, with a comparison to the standard EKF approach.

This paper is organised as follows: The recursive Bayesian estimation framework of particle filtering is presented, followed by the hypothesis testing paradigm. The aircraft, sensor, and noise models are then presented. Two icing detection approaches are then proposed, based on a Gaussian kernel for icing probability evaluation, then using a discrete jump model. The icing effects are then modelled, which is followed by a description of the EKF used for comparison. A simulation analysis is then presented, with a comparison between the Gaussian kernel and discrete jump particle filters and the EKF under moderate and severe icing. The comparison is performed in terms of parameter estimation accuracy, ice detection delay, and the smoothness and reliability of the icing probability computation. In the presence of icing, moderate to severe distortions of the sensor noise characteristics are both evaluated. The uniform noise simulations were used to test a worst-case measurement error scenario. The simulations with a Gaussian mixture noise were used to evaluate the effects of more moderate but likely distorted sensor noise characteristics due to icing. Both particle filters achieve a higher state and parameter estimation accuracy, with improved ice detection reliability, compared to a standard EKF. The discrete jump PF is shown to be particularly suited to the detection of abrupt icing events, while the Gaussian kernel PF efficiently handles the detection of more gradual onsets of aircraft icing. A dual framework can therefore be developed from these two approaches.

## 2. Recursive Bayesian Estimation and Particle Filtering

In this section, we provide a brief overview of recursive Bayesian estimation, then introduce the PF for nonlinear, non-Gaussian systems.

### 2.1. Recursive Bayesian Estimation

The goal of recursive Bayesian estimation or filtering is to estimate the posterior distribution of the state $x_{k}$ at time *k*, given all observations up to point $z_{1:k}$, noted as $p(x_{k}|z_{1:k})$, based on Bayesian inference under the Markov assumption.All of these filters consist of two main steps: a prediction step based on the system dynamics, and a correction step based on current measurements.

The Bayes filter is the most generic case and is mostly used for discrete systems with a finite number of states. The Kalman filter is considered an analytical solution of the Bayes filter under the assumption of linear transition and measurement models, with Gaussian noise. The PF, on the other hand, is an approximation of the solution with minimal restrictions on the model or noise type.

### 2.2. The Particle Filter

The particle filter, also known as the Sequential Monte Carlo (SMC) method, is a nonparametric implementation of the Bayes filter. It is particularly suitable for nonlinear and non-Gaussian systems where analytical solutions are intractable.

In particle filtering, the posterior distribution is approximated using a set of weighted particles ${\left\{x_{k}^{\left(i\right)},w_{k}^{\left(i\right)}\right\}}_{i=1}^{N}$:

(1) $$p\left(x_{k}|z_{1:k}\right)\approx \sum_{i=1}^{N}w_{k}^{\left(i\right)}\;\delta \left(x_{k}-x_{k}^{\left(i\right)}\right)$$

where δ(·) is the Dirac delta function, $x_{k}^{\left(i\right)}$ is the *i*-th particle, and $w_{k}^{\left(i\right)}$ is its associated weight.

#### 2.2.1. Importance Sampling

Importance sampling is a method first introduced in [12] to approximate expectations of a probability distribution when direct sampling is difficult. Instead of sampling directly from the target distribution (posterior in this case), particles are drawn from a proposal distribution $q(x_{k}|x_{k-1},z_{k})$ that is easy to sample from:

$$x_{k}^{\left(i\right)}∼q\left(x_{k}|x_{k-1}^{\left(i\right)},z_{k}\right)$$

Then, the integration for computing the expectation is approximated by a discrete summation using weights assigned to the particles. By taking the proposal distribution to be equal to the prior transition model $p(x_{k}|x_{k-1})$, the weights are computed using the following recursive importance sampling equation [13]:

(2) $$w_{k}^{\left(i\right)}=\frac{w_{k-1}^{\left(i\right)}\;p\left(z_{k}|x_{k}^{\left(i\right)}\right)}{\sum_{j=1}^{N}w_{k-1}^{\left(j\right)}\;p\left(z_{k}|x_{k}^{\left(j\right)}\right)}$$

#### 2.2.2. The Degeneracy Problem and Resampling

A major challenge in particle filtering is particle degeneracy, where after several iterations, most particles carry negligible weights. To combat this, a resampling step is introduced.

The level of degeneracy of a set of particles can be quantified using the effective sample size [14]:

(3) $$N_{\mathrm{eff}}=1/\sum_{i=1}^{N}{\left(w_{k}^{\left(i\right)}\right)}^{2}$$

When $N_{\mathrm{eff}}<N_{\mathrm{thr}}$, where $N_{\mathrm{thr}}\in \left[1,N\right]$, resampling is triggered to discard low-weight particles and duplicate high-weight ones, thereby maintaining particle diversity [15].

When such a condition is encountered, the resampling algorithm is applied. Several resampling strategies exist, including multinomial resampling, residual resampling [16], stratified resampling [17], and systematic resampling [18]. Choosing an appropriate resampling technique is crucial for maintaining particle diversity and improving the estimation accuracy.

The more general particle filtering algorithms, including initialisation, state propagation (prediction), measurement update with weight computation, sequential importance resampling (SIR), and state estimation, are described in [15,18].

## 3. Hypothesis Testing in Statistical Decision Theory

Statistical decision theory is a mathematical framework for making rational decisions under uncertainty, combining statistical models with loss functions to quantify decision consequences based on observations.

The hypothesis testing procedure formalises decision-making between two competing hypotheses (or more) through the following components:

- Test Statistic *T*: Function of observations *x* (e.g., T(x)=LRT).
- Decision Rule: Threshold comparison (Reject $H_{0}$ if T(x)>κ).
- Performance Metrics: False alarm rate ($\bar{\alpha }$), Detection power ($1-\bar{\beta }$).

This paradigm is particularly effective for fault detection, where we must distinguish between nominal and abnormal system states.

The first step of this approach is to construct a statistical model with certain parameters θ to represent certain characteristics of the system. These parameters have specific values $\theta_{0}$ in the null hypothesis, which is the case of the nominal system. These parameters change when another hypothesis occurs, called the alternative hypothesis, such that, in our case, the following are used:

- Null Hypothesis $H_{0}$: $\theta =\theta_{0}$ (No icing).
- Alternate Hypothesis $H_{c}$: $\theta \neq \theta_{0}$ (Icing).

In our case, θ represents the set of aerodynamic parameters $C_{i_{j}}$, i∈{L,D,Y,l,m,n} and $j\in \{0,\alpha ,q,\delta_{e}\}$. The null hypothesis refers to the clean condition, and the alternate hypothesis to icing condition.

The second step consists of performing a hypothesis test to decide between the two hypotheses. For this, three strategies exist in the statistical literature: the first is based on the so-called *Wald scores*, the second is a *likelihood ratio test*, and the third is based on measure of *drop in dispersion* [19]. The most commonly used method is the likelihood ratio test, which computes the ratio of the likelihood of the signal under the two different hypotheses:

$$LRT=\frac{p(x|H_{c})}{p(x|H_{0})}$$

The signal *x* can be any signal representing the system’s behavior: for example, residual, observations, or state estimate. Some other commonly used statistical tests described in [20] are the cumulative sum algorithm (CUSUM) [21], which sums the log of LRTs, and the sequential probability ratio test (SPRT) [22]. However, all of these tests need the parameters of the two distributions to be known.

When the distribution function parameters are unknown in one or both hypotheses, the maximum likelihood estimation (MLE) is used to estimate these parameters given a sequence of data $\left\{x_{1},\ldots ,x_{n}\right\}$. The generalised likelihood ratio test (GLRT) is then applied using these estimates.

$$GLRT=\frac{p(x|{\hat{\theta }}_{c},H_{c})}{p(x|{\hat{\theta }}_{0},H_{0})}$$

However, GLRT requires the form of the PDF to be known. The PDF is often taken as the normal distribution, but many systems are non-Gaussian. To overcome such constraints, particle filtering-based methods have been used. Within an SMC framework, particles can be used to approximate any distribution by a discrete distribution. Particle filters are also used for the general nonlinear case since GLRT assumes a linear system.

In our first approach, the observation likelihood (OL) test [23] will be used, and for the second approach, a simple discrete jump model will be applied on a PF.

## 4. Aircraft State and Measurement Models Used by the Estimation Algorithms

This section presents the models used for the PF-based icing detection and estimation of aerodynamic parameter changes ($\Delta C_{D}$, $\Delta C_{L}$). By explicitly incorporating aerodynamic degradation into the state space, the method estimates airspeed, angle of attack (AoA), and parameter changes using pitot-tube and accelerometer measurements. Unknown parameter dynamics are handled via a Random Walk with particle selection.

Two detection approaches are proposed. The first uses the complement of the Gaussian kernel to compute icing probability from $\Delta {\hat{C}}_{D}$ and $\Delta {\hat{C}}_{L}$ deviations, with sensitivity controlled by tuning standard deviation parameters.

The second approach employs statistical decision theory using a particle subset to test the icing hypothesis with guided noise generation. Constraints ($\Delta C_{D}\geq 0$, $\Delta C_{L}\leq 0$) guide Gamma-distributed process noise generation. A discrete jump indicator $J_{t}$ is added to specify icing occurrence ($J_{t}=1$) or not ($J_{t}=0$). Noise is introduced only during jump events; otherwise, $\Delta C_{D}=\Delta C_{L}=0$. A randomly selected particle subset is assigned to jump events with noise generation. If icing is present, these particles dominate after resampling due to higher likelihood, enabling full parameter set estimation.

### 4.1. Transition Model

The transition model used for the PF is given by:

(4) $$x_{t}=x_{t-1}+f\left(x_{t-1},u_{t}\right)\Delta t+v_{t}$$

where the state to be estimated consists of the airspeed $V_{a}$ and AoA denoted α, as well as the change in the drag and lift coefficients $\Delta C_{D}$ and $\Delta C_{L}$, respectively:

(5) $$x={\left[\begin{array}{cccc} V_{a} & \alpha & \Delta C_{D} & \Delta C_{L} \end{array}\right]}^{T}$$

and the control input and function *f* are defined as:

(6) $$u={\left[\begin{array}{ccc} \theta & q & \delta_{e} \end{array}\right]}^{T},\;\dot{x}=f\left(x,u\right)$$

with $v_{t}$ process noise under the null hypothesis, sampled from any distribution (uniform and mixed Gaussian distributions will be tested in simulation), *u* control inputs for the filter assumed to be available, and *f* represents the state dynamics.

The dynamics of $x_{1}$ and $x_{2}$ are given by:

(7) $$\begin{array}{cc} {\dot{V}}_{a} & =\frac{1}{m}\left(F_{x}\cos\alpha +F_{z}\sin\alpha \right) \end{array}$$

(8) $$\begin{array}{cc} \dot{\alpha } & =\frac{1}{mV_{a}}\left(-F_{x}\sin\alpha +F_{z}\cos\alpha \right)+q \end{array}$$

Such that:

(9) $$\begin{array}{cc} F_{x} & =-\frac{1}{2}\rho V_{a}^{2}S\left(C_{D}\cos\alpha -C_{L}\sin\alpha \right)+F_{T}-mg\sin\theta \end{array}$$

(10) $$\begin{array}{cc} F_{z} & =-\frac{1}{2}\rho V_{a}^{2}S\left(C_{D}\sin\alpha +C_{L}\cos\alpha \right)+mg\cos\theta \cos\phi \end{array}$$

where $C_{D}$ and $C_{L}$ are the true drag and lift coefficients, which are computed based on the estimates of $\Delta C_{D}$ and $\Delta C_{L}$, as follows:

(11) $$\begin{array}{cc} \Delta C_{L} & =C_{L}-C_{L_{\exp}},\\ \Delta C_{D} & =C_{D}-C_{D_{\exp}}. \end{array}$$

where the subscript “exp” denotes the expected values (nominal, clean-airframe), and no subscript refers to the actual value during flight. For a fixed-wing UAV operating in steady, clean flight conditions, the expected aerodynamic coefficients are given as functions of the longitudinal states [24]. We use the following linear approximation:

(12) $$\begin{array}{cc} C_{L_{\exp}}\left(\alpha ,q,\delta_{e}\right) & =C_{L_{0}}+C_{L_{\alpha }}\alpha +C_{L_{q}}\frac{c}{2V_{a}}q+C_{L\delta_{e}}\left|\delta_{e}\right| \end{array}$$

(13) $$\begin{array}{cc} C_{D_{\exp}}\left(\alpha ,q,\delta_{e}\right) & =C_{D_{0}}+C_{D_{\alpha }}\alpha +C_{D_{q}}\frac{c}{2V_{a}}q+C_{D\delta_{e}}\left|\delta_{e}\right| \end{array}$$

The linear approximation is justified for the operational envelopes considered in the analysis, where the angle of attack remains within the pre-stall linear lift region before and shortly after the onset of icing; the control surface deflections are moderate and pitch rate remains small compared to the freestream velocity. It is important to ensure that algorithms detect ice shortly after the onset of icing for the model to remain valid until ice is detected. The variation in the aerodynamic coefficients is assumed to be primarily attributed to icing effects, under the hypothesis that no other major faults (e.g., engine failure or structural damage) are present.

### 4.2. Measurement Model

Without direct measurement of the AoA, estimates of $\Delta C_{D}$ and $\Delta C_{L}$ may not be accurate. To address this issue, we employ an alternative method based on wind estimation to estimate the AoA. This method is proposed and explained in the book [24], Chapter 8, Section 7. We then use it as a measurement and estimate it again to improve accuracy (the AoA estimate from the wind will be quite noisy in practice).

The measurement model is given by:

(14) $$z_{t}=h\left(x_{t},u_{t}\right)+w_{t}$$

with $w_{t}$ measurement noise, where the measurement function *h* is:

$$h={\left[\begin{array}{cccc} V_{a}^{m} & \alpha^{m} & a_{x}^{m} & a_{z}^{m} \end{array}\right]}^{T}$$

such that $\alpha^{m}=\arctan\left({\hat{w}}_{r}/{\hat{u}}_{r}\right)$ is the AoA estimate from the wind, and $V_{a}^{m}$ is the airspeed reconstructed from the pitot–static tube after low-pass filtering (LPF):

$$V_{a}^{m}=\sqrt{\frac{2}{\rho }\mathrm{LPF}\left(y_{\mathrm{pitot}}\right)}$$

and $a_{x}^{m}$ and $a_{z}^{m}$ are accelerometer measurements (excluding gravitational acceleration):

(15) $$\begin{array}{cc} a_{x}^{m} & =-\frac{\rho V_{a}^{2}S}{2m}\left(C_{D}\cos\alpha -C_{L}\sin\alpha \right)+\frac{F_{T}}{m} \end{array}$$

(16) $$\begin{array}{cc} a_{z}^{m} & =-\frac{\rho V_{a}^{2}S}{2m}\left(C_{D}\sin\alpha +C_{L}\cos\alpha \right) \end{array}$$

### 4.3. Random Walk with Noise Selection

To estimate $\Delta C_{D}$ and $\Delta C_{L}$, we use a Random Walk combined with particle filtering, which allows selection of the added noise. For each particle, we sample noise from any distribution with zero mean and finite known variance, for $\Delta C_{D}$ and $\Delta C_{L}$, as follows:

(17a) $$\varepsilon_{D}^{\left(i\right)}\left(k\right)∼W_{D}\left(\Theta_{D}\right)$$

(17b) $$\varepsilon_{L}^{\left(i\right)}\left(k\right)∼W_{L}\left(\Theta_{L}\right)\;\;$$

The normal distribution is the ideal sensor noise characteristic for Kalman filters, but in the presence of icing, moderate and severe distortions with respect to the ideal normal distribution need to be considered. A Gaussian mixture noise is therefore considered in simulations to test the effect of moderate distortions with respect to the normal distribution. A uniform distribution will also be tested to evaluate a form of worst-case distortion of the sensor noise characteristic due to icing. Ice accretion can lead to deviations from the Gaussian noise assumption by causing pitot–static probe obstructions and by accumulating on external sensors such as GPS antennas.Icing can also indirectly affect the inertial navigation system measurements in UAVs under cold temperatures and by altering the aerodynamic forces and dynamics of the aircraft. The sensor characteristics under consideration have a zero mean. Sensor faults can change the mean, but this is beyond the scope of the paper, even if the proposed filters can be adapted to situations with a non-zero mean or more asymmetric noise characteristics.

It is noteworthy that the likelihood equation for $p(z_{k}|x_{k})$ is dependent on the noise distribution (see [25] for the Gaussian mixture likelihood and [26] for the uniform likelihood). The noise distribution type therefore determines the likelihood function used in the importance sampling Equation (2).

Then, a Random Walk is used for propagation, which adds noise to the previous value:

(18a) $$\Delta C_{D}^{\left(i\right)}\left(k\right)=\Delta C_{D}^{\left(i\right)}\left(k-1\right)+\varepsilon_{D}^{\left(i\right)}\left(k\right)$$

(18b) $$\begin{array}{cc} \Delta C_{D}^{\left(i\right)}\left(k\right) & =\Delta C_{D}^{\left(i\right)}\left(k-1\right)+\varepsilon_{L}^{\left(i\right)}\left(k\right) \end{array}$$

This relation appears to be random, but thanks to resampling, it allows for the selection of the ’right’ amount of noise to be added for obtaining values close to the real ones. With a sufficiently large number of particles, the drawn samples $\varepsilon_{D}^{\left(j\right)}\left(k\right)$ and $\varepsilon_{L}^{\left(j\right)}\left(k\right)$ in promising regions give relatively higher likelihood when α and $V_{a}$ are accurate (which will be the case since they are directly measured). This helps move in the right direction and select the right amount of noise to be added. These particles will dominate after resampling, which updates the estimate to a value close to its real value.

## 5. Icing Detection

### 5.1. Gaussian Kernel for Computing Icing Probability

In this method, the probability of icing is computed using a complement of a 2D Gaussian distribution; the higher the deviation of $\Delta C_{D}$ and $\Delta C_{L}$ from 0, the higher the probability:

(19) $$p_{ice}=1-\exp\left(-\frac{1}{2}\left(\frac{\Delta C_{D}^{2}}{\sigma_{2}^{2}}+\frac{\Delta C_{L}^{2}}{\sigma_{1}^{2}}\right)\right)$$

The values of $\sigma_{1}$ and $\sigma_{2}$ should be tuned accordingly to have the desired sensitivity. Icing is detected when $p_{ice}$ is greater than a certain probability, for instance, 0.95.

### 5.2. Discrete Jump Model

The proposed discrete jump method combines hypothesis testing based on a Bernoulli process model with particle filtering to decide whether icing is present or not. The jump hypothesis is not independent across particles, and its occurrence is reinforced by measurement likelihoods and resampling.

In addition to the drag ($\Delta C_{D}$) and lift ($\Delta C_{L}$) variations, a binary jump indicator $J_{t}$ is estimated as part of the state vector. Although the dynamics of $\Delta C_{D}$ and $\Delta C_{L}$ are unknown, their signs are constrained: since icing causes an increase in the drag coefficient and a decrease in the lift coefficient, the sign of their variations is always constant: $\Delta C_{L}\leq 0$, $\Delta C_{D}\geq 0$. These constraints guide noise generation to test the change hypothesis. The null and alternative hypotheses (change) are formulated as follows:

(20) $$\begin{array}{cc} H_{0}:\Delta C_{L}=\Delta C_{D}=0 & \end{array}$$

(21) $$\begin{array}{c} H_{c}:\Delta C_{L}<0,\Delta C_{D}>0 \end{array}$$

However, to avoid having false alarms when $\Delta C_{L}$ or $\Delta C_{D}$ is small, we formulate the following decision problem:

(22) $$\begin{array}{cc} H_{0}:\Delta C_{L}=\Delta C_{D}=0 & \end{array}$$

(23) $$\begin{array}{c} H_{c}:\Delta C_{L}<\kappa_{L},\Delta C_{D}>\kappa_{D} \end{array}$$

For some thresholds, $\kappa_{L}<0$ and $\kappa_{D}>0$. Increasing the thresholds favours a reduction in false alarms while reducing them favours a reduction in missed detections.

Noise is introduced only when a jump is hypothesised; otherwise, $\Delta C_{D}=\Delta C_{L}=0$. A small probability $p_{c}$ is used to randomly assign the jump hypothesis to a small subset of particles. When the jump hypothesis yields significantly higher likelihoods, even when initially supported by few particles, those particles accumulate large weights and are duplicated during resampling. As a result, the majority (or all) of the subsequent particles accept the jump hypothesis ($J_{t}=1$).

In this case, the hypothesis is accepted globally, and the estimation of $\Delta C_{D}$ and $\Delta C_{L}$ proceeds across the entire particle set (since they are no longer close to zero). The dynamics used for their estimation are based on the Random Walk, using the uniform distribution U.

For the noise generated during the hypothesis testing procedure, we propose to use the Gamma distribution Γ to sample positive noise for $\Delta C_{D}$ and $-\Delta C_{L}$.

Thus, if J=0, then $\Delta C_{D}=\Delta C_{L}=0$. Otherwise, if J=1, then the hypothesis is tested by using:

(24) $$\Delta C_{D}^{\left(i\right)}∼\Gamma \left(k_{0},\theta_{0}\right),$$

and

(25) $$\Delta C_{L}^{\left(i\right)}∼-\Gamma \left(k_{0}^{\prime },\theta_{0}^{\prime }\right),$$

for the subset of the randomly chosen particles with a certain probability $p_{c}$. The pseudo-algorithm of this approach is shown in Algorithm 1.
**Algorithm 1** Discrete jump model for $\Delta C_{D}$ and $\Delta C_{L}$.**Require:** $N_{\mathrm{particles}},\;p_{c},\;k_{0},\;\theta_{0},\;c$**Ensure:** Updated particles matrix **for** 
i=1 **to** $N_{\mathrm{particles}}$ **do**
       $x_{\mathrm{prev}}\leftarrow \mathrm{particles}\left[i,3:4\right]$▹ Previous position       $J_{\mathrm{prev}}\leftarrow \mathrm{particles}\left[i,5\right]$▹ Previous jump status       **if** $J_{\mathrm{prev}}=0$ **then**▹ No jump yet             **if** $\mathrm{rand}\left(\right)<p_{c}$ **then**▹ Propose jump with probability $p_{c}$               $x_{\mathrm{new}}\leftarrow \Gamma \left(k_{0},\theta_{0}\right)$▹ Sample initial jump               $J_{\mathrm{new}}\leftarrow 1$▹ Update jump status             **else**               $x_{\mathrm{new}}\leftarrow 0$▹ No movement               $J_{\mathrm{new}}\leftarrow 0$▹ Maintain jump status             **end if**       **else**▹ Post-jump phase             Δx←U(−c,c)▹ Sample drift + noise             $x_{\mathrm{new}}\leftarrow x_{\mathrm{prev}}+\Delta x$▹ Update position             $J_{\mathrm{new}}\leftarrow 1$▹ Maintain jump status       **end if**       $\mathrm{particles}\left[i,3:5\right]\leftarrow \left[x_{\mathrm{new}},J_{\mathrm{new}}\right]$▹ Update particle state **end for**

In the Algorithm 1, we use $x_{prev}={\left[\begin{array}{cc} \Delta C_{D}\left(t-1\right) & \Delta C_{L}\left(t-1\right) \end{array}\right]}^{T}$.

The mean and variance of samples drawn from a Gamma distribution Γ(k,θ) are given by:

(26) $$\begin{array}{cc} E[x] & =k\theta \end{array}$$

(27) $$\begin{array}{cc} V[x] & =\mathrm{Var}\left(x\right)=k\theta^{2} \end{array}$$

which is useful for computing thresholds and tuning $k_{0}$, $k_{0}^{\prime }$, $\theta_{0}$, and $\theta_{0}^{\prime }$.

This method has some similarities with the work in [27], which adapts noise generation in a PF using the Liu and West filter [28] with adaptive noise added on the variance, and accelerates its learning using an equation from the genetic algorithm. Particles with high likelihood automatically replace low-weight particles using the resampling mechanism. Both stochastic volatility (gradual changes) and regime shift (sudden jumps) were evaluated using this method.

Statistic used for Detection

The proportion of particles having $J_{t}=1$ serves as an empirical estimate of the jump likelihood and is used as an indicator for icing detection:

(28) $$p_{icing}\left(t\right)=p_{jump}\left(t\right)=\frac{\sum_{i:J_{t}^{\left(i\right)}=1}}{N}$$

The icing occurrence time $t_{occ}$ is such that $p_{icing}\geq p_{d}$ with $p_{d}$ being some preset detection probability (for example, 95%). Note that $t_{occ}$ is sensitive to $p_{c}$, and a high value of $p_{c}$ can lead to a higher false alarm rate due to measurement and modelling errors. Such false alarms can be overcome by adequate tuning, and performing a check on the environment temperature if available.

## 6. Icing Effect Model

To model the icing effect on the UAV’s aerodynamics, we use the popular model by Bragg et al. [2,9,29]:

$$C_{i}^{iced}=C_{i}^{clean}\left(1+\eta_{ice}K_{i}\right)$$

where $C_{i_{j}}^{iced}$ is an aerodynamic parameter under icing conditions, $C_{i_{j}}^{clean}$ is its nominal value, $\eta_{ice}$ is the icing severity factor, and $K_{i}$ is a constant that reflects how strongly the aerodynamic coefficient is affected by icing.

Here, i∈{L,D,m} with $j\in \{0,\alpha ,q,\delta_{e}\}$.

The icing severity factor is supposed to vary slowly since icing accretion takes some time to occur. Its dynamics can be represented simply by a saturation function, or using the following equations [9]:

$$\begin{array}{cc} {\dot{\eta }}_{ice} & =N_{1}\left(1+N_{2}\eta_{ice}\right)d_{\eta }\\ d_{\eta }\left(t\right) & =\frac{1}{2}\left(1-\cos\left(\frac{2\pi }{T_{cld}}\right)\right)\\ N_{1}\left(t\right) & =\frac{1}{N_{2}T_{cld}}\ln\left(1+N_{2}r_{1}\right)\\ N_{2}\left(t\right) & =\frac{r_{1}-2r_{2}}{r_{2}^{2}} \end{array}$$

with $t_{occ}\leq t\leq t_{occ}+T_{cld}$, where $t_{occ}$ is the accretion time, $T_{cld}$ denotes the accretion duration, and $r_{1}$ and $r_{2}$ are accretion rates (value of η) at $t_{occ}$ and $t_{occ}/2$, respectively. In our simulation, we have also included a variation of the UAV mass and wing surface:

(29) $$\begin{array}{cc} m(t) & =m_{0}+\eta \left(t\right)K_{m} \end{array}$$

(30) $$\begin{array}{cc} S(t) & =S_{0}+\eta \left(t\right)K_{S} \end{array}$$

where $m_{0}$ and $S_{0}$ are the nominal mass and wing surface, respectively, $K_{m}$ is the maximum accreted mass, and $K_{S}$ is the maximum added surface by icing.

## 7. Extended Kalman Filter for Comparison

The EKF is only used for comparison purposes and is therefore briefly described in this section. The state and measurement vectors are the ones previously defined in Equations (5) and (6).

The linearised state transition matrix is obtained by Jacobian linearisation using $A=\frac{\partial \dot{x}}{\partial x}$:

$$A=\left[\begin{array}{cccc} A_{11} & A_{12} & A_{13} & A_{14}\\ A_{21} & A_{22} & A_{23} & A_{24}\\ 0 & 0 & 0 & 0\\ 0 & 0 & 0 & 0 \end{array}\right]$$

The elements of the first row of A are given by:

(31) $$\begin{array}{cc} A_{11} & =\frac{1}{m}\left(\frac{\partial F_{x}}{\partial V_{a}}\cos\alpha +\frac{\partial F_{z}}{\partial V_{a}}\sin\alpha \right) \end{array}$$

(32) $$\begin{array}{cc} A_{12} & =\frac{1}{m}\left(\frac{\partial F_{x}}{\partial \alpha }\cos\alpha -F_{x}\sin\alpha +\frac{\partial F_{z}}{\partial \alpha }\sin\alpha +F_{z}\cos\alpha \right) \end{array}$$

(33) $$\begin{array}{cc} A_{13} & =-\frac{\bar{q}S}{m}=-\frac{\rho V_{a}^{2}S}{2m} \end{array}$$

(34) $$\begin{array}{cc} A_{14} & =0 \end{array}$$

The elements of the second row of A are given by:

(35) $$\begin{array}{cc} A_{21} & =\frac{1}{mV_{a}}\left(-\frac{\partial F_{x}}{\partial V_{a}}\sin\alpha +\frac{\partial F_{z}}{\partial V_{a}}\cos\alpha \right)-\frac{1}{mV_{a}^{2}}\left(-F_{x}\sin\alpha +F_{z}\cos\alpha \right) \end{array}$$

(36) $$\begin{array}{cc} A_{22} & =\frac{1}{mV_{a}}\left(-\frac{\partial F_{x}}{\partial \alpha }\sin\alpha -F_{x}\cos\alpha +\frac{\partial F_{z}}{\partial \alpha }\cos\alpha -F_{z}\sin\alpha \right) \end{array}$$

(37) $$\begin{array}{cc} A_{23} & =0 \end{array}$$

(38) $$\begin{array}{cc} A_{24} & =-\frac{\bar{q}S}{mV_{a}}=-\frac{\rho V_{a}S}{2m} \end{array}$$

The control matrix B is obtained by a similar Jacobian linearisation of the function f(x,u) with respect to the control vector of Equation (6).

The measurement Jacobian matrix is given by $H=\frac{\partial h}{\partial x}$ as a 4×4 matrix:

$$H=\left[\begin{array}{cccc} \frac{1}{m}\frac{\partial F_{x}}{\partial V_{a}} & \frac{1}{m}\frac{\partial F_{x}}{\partial \alpha } & -\frac{\bar{q}S\cos\alpha }{m} & \frac{\bar{q}S\sin\alpha }{m}\\ \frac{1}{m}\frac{\partial F_{z}}{\partial V_{a}} & \frac{1}{m}\frac{\partial F_{z}}{\partial \alpha } & -\frac{\bar{q}S\sin\alpha }{m} & -\frac{\bar{q}S\cos\alpha }{m}\\ 1 & 0 & 0 & 0\\ 0 & 1 & 0 & 0 \end{array}\right]$$

where the dynamic pressure is

$$\bar{q}=\frac{1}{2}\rho V_{a}^{2},$$

The continuous-time state transition and control matrices are discretised using a first-order Euler approximation:

$$\begin{array}{cc} A_{d} & =I_{4}+\Delta t\;A,\\ B_{d} & =\Delta t\;B \end{array}$$

where Δt is the sampling time period. For the numerical simulation analysis, the EKF is subjected to the same process and measurement noises as the particle filters and to the same initial uncertainty on the state.

The icing probability of the EKF is again computed using equation (19) and is a function of a squared Mahalanobis distance representing the sum of the squared normalised deviations of the lift and drag coefficients ΔCL and ΔCD relative to their standard deviations. The icing probability increases when this Mahalanobis distance increases.

A Bernoulli process-based jump logic is then used to declare icing when this probability exceeds a confidence threshold of 0.95.

## 8. Simulation Results

We have simulated sensors with noisy measurements for a more realistic scenario. In the simulations for the three methods (Gaussian kernel PF, discrete jump PF, and EKF), the following values of noise parameters were taken:

- Process standard variation: $\sigma_{V_{a}}^{p}=0.2\;m/s$, $\sigma_{\alpha }^{p}=0.005\;\mathrm{rad}\approx 0.28{}^{\circ}$, $\sigma_{\Delta C_{L}}=\sigma_{\Delta C_{D}}=0.005$.
- Sensor noise: $\sigma_{ax}=0.5\;m/s^{2}$, $\sigma_{ax}=0.5\;m/s^{2}$.
- Simulated noise on $\hat{\alpha }$ and ${\hat{V}}_{a}$: $\sigma_{\alpha }=0.014\;\mathrm{rad}\approx 0.8{}^{\circ}$ and $\sigma_{Va}=1\;m/s$, respectively.

Different sensor noise characteristics were considered for the analysis, including a zero average uniform distribution, as ∼U(−σ,σ) for each sensor to test a worst-case sensing scenario and a Gaussian-mixture noise representing more realistic and moderate sensor imperfections in the presence of ice accretion. The sensor and process noise parameters were based on realistic scenarios from [24]. The effect of increasing the process noise was evaluated but not included for brevity and because it did not alter the main findings on the characteristics (smoothness, rapidity) of the icing probability estimation. The comparison is therefore more focused on the prominent effects of icing severity and the types of the noise distributions.

For the Gaussian mixture noise simulation, a mixture of two zero mean Gaussians weighted as 0.9 and 0.1, respectively, with different standard deviations, was considered for all states except the noise distributions of $C_{L}$ and $C_{D}$, which are modelled as gamma functions when a jump is hypothesised to allow the modelling of more abrupt aerodynamic changes. The mixed Gaussian noise equation can be written compactly as:

$$v_{k}∼w\;N\left(0,\Sigma_{1}\right)+\left(1-w\right)\;N\left(0,\Sigma_{2}\right)$$

where $\Sigma_{1}$ represents the previously provided sensor standard deviations and $\Sigma_{2}=2\Sigma_{1}$ represents a higher uncertainty (larger distribution tail) linked to icing.

The PF is not tied to a noise type, unlike the Kalman filter, which is only optimised for the normal Gaussian law, not for mixed-Gaussian noise.

We have tested the methods under both moderate and severe icing conditions. Icing occurs at $t_{occ}=80\;s$ and lasts for 120 s for severe icing, and 200 s for moderate icing (until η is saturated at 1). We used 1000 particles for the PF. Accretion rates were taken as follows: $r_{1}=1$, $r_{2}=0.7$. The sampling time period is given by Δt=0.05s.

The icing detection performance of the PF using the Gaussian Kernel approach of Section 5.1 and the discrete jump model of Section 5.2 is evaluated using numerical comparisons and compared to the performance of the EKF.

### 8.1. First Approach: Gaussian Kernel

**Figure 1:**
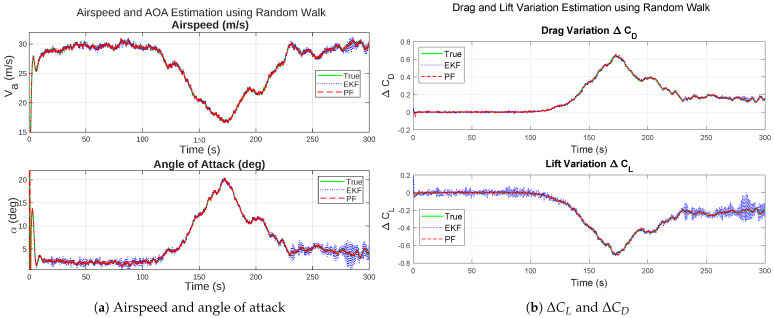
Comparative results under moderate icing for the Gaussian kernel PF and EKF, uniform noise.

**Figure 2:**
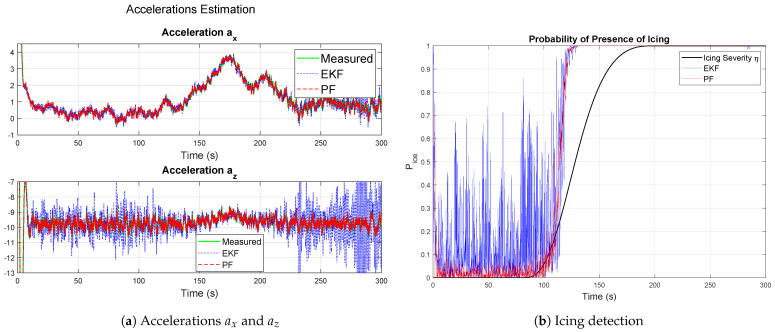
Comparative results under moderate icing for the Gaussian kernel PF and EKF, uniform noise.

The state estimation and icing detection estimation results of the Gaussian kernel-based PF and the EKF under a uniform noise are illustrated in Figure 1 for the airspeed, angle of attack and aerodynamic coefficients, and Figure 2 for the icing probability and the acceleration estimates.

In Figure 1, the state estimation of the Gaussian Kernel PF clearly outperforms the EKF, especially for the maximum error deviations of the lift coefficient deviations and the angle of attack. Following the icing onset at $t_{occ}=80\;s$, both coefficients show smooth and gradual changes consistent with the simulated aerodynamic degradation. The estimated trajectories of the Gaussian kernel PF exhibit limited fluctuations, mainly attributed to measurement noise and finite sampling in the particle representation. The close alignment between true and estimated profiles indicates that the Random Walk model and the particle filtering framework effectively capture slow aerodynamic parameter drifts under moderate icing. The EKF estimates are visibly more noisy towards the end of the simulation.

In Figure 2, the icing probability of the Gaussian kernel PF increases progressively after the occurrence of icing, crossing the detection confidence threshold of 0.95 shortly after the onset of icing delay. The z-axis acceleration and icing probability estimates of the EKF have much larger fluctuations before the average of the icing probability starts increasing. The smoother Gaussian kernel PF characteristic is attributed to its conservative sensitivity, controlled through the kernel parameters $\sigma_{1}$ and $\sigma_{2}$, which were tuned to limit false alarms. The smooth rise of $p_{ice}$ using the Gaussian kernel PF corresponds closely with the simulated icing severity profile, confirming the model’s ability to reliably track gradual ice accretion dynamics.

**Figure 3:**
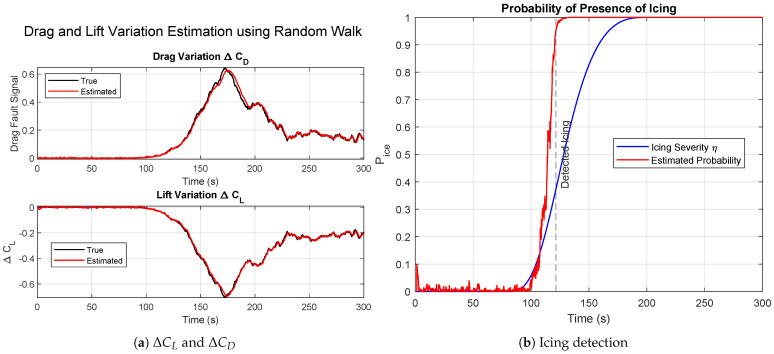
Results for severe ice using the Gaussian kernel PF.

The Gaussian kernel PF under severe icing conditions is shown in Figure 3 to estimate the icing probability even more significantly accurately than expected. In this scenario, the aerodynamic degradation occurs more abruptly, with larger magnitudes of $\Delta C_{L}$ and $\Delta C_{D}$. The estimates follow the true variations closely, maintaining stability despite the increased rate of change. The corresponding icing probability reaches the detection threshold more rapidly than in the moderate case, due to the higher deviations in aerodynamic coefficients that amplify the likelihood update in (2). The results demonstrate that the PF maintains consistent estimation quality across varying icing severities, provided that the process and measurement noise variances remain within realistic limits.

**Figure 4:**
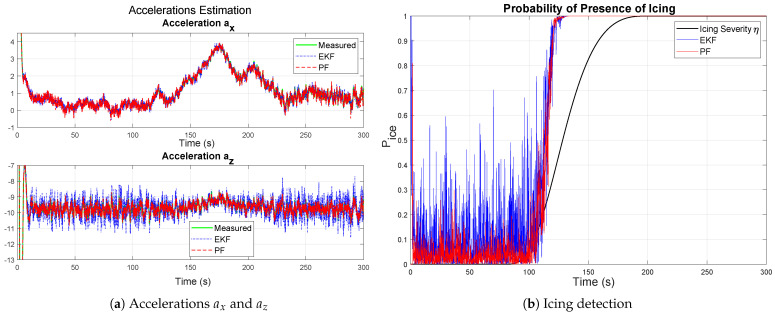
Results for moderate icing using the Gaussian kernal PF, Gaussian mixture noise.

For moderate icing conditions in the case of mixed-Gaussian noise, the body acceleration estimation and icing detection results are provided in Figure 4 with a comparison to the EKF. The Gaussian kernel-based PF outperforms the EKF in terms of estimation accuracy for the Z-axis acceleration and for the icing probability.

**Figure 5:**
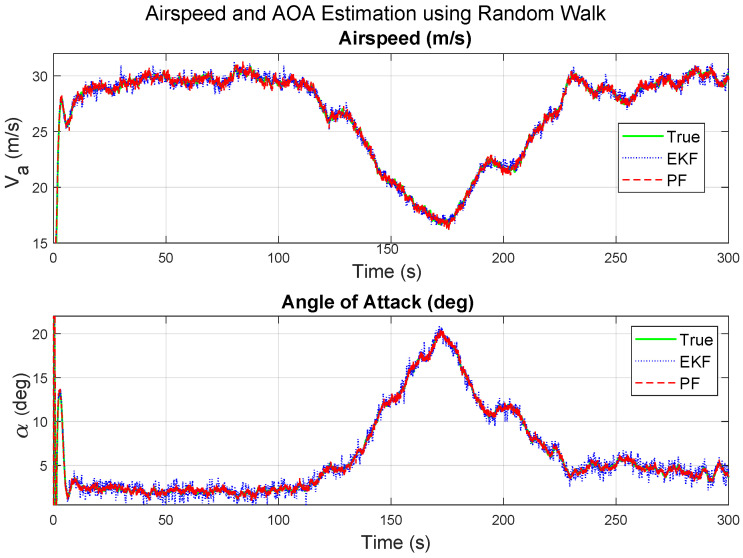
Airspeed and angle of attack. Results for moderate icing using the Gaussian kernel PF with Gaussian mixture noise.

Under the same Gaussian mixed noise characteristic, the speed and angle of attack estimates of the Gaussian kernel PF are also more accurate than those of the EKF, as shown in Figure 5.

In the remainder of this paper, the figures represent filter performance under uniform noise for consistency, and comparisons of the second PF approach to the EKF are summarised in tables for brevity.

### 8.2. The Second Approach: Discrete Jump Model

**Figure 6:**
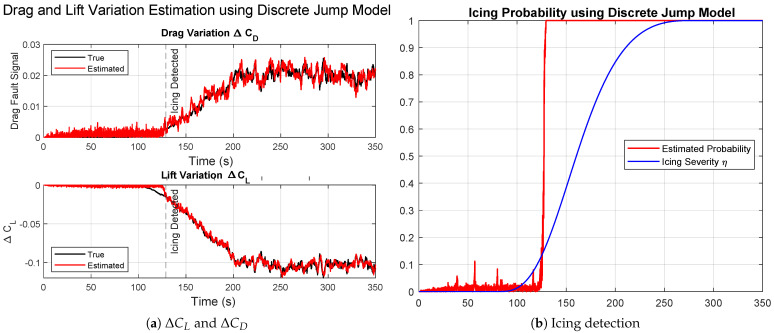
Results for moderate ice using the second approach, discrete jump model.

Figure 6 presents the results obtained with the discrete jump model in the case of moderate icing.

Figure 6a shows the time evolution of $\Delta C_{L}$ and $\Delta C_{D}$. The introduction of the binary jump indicator $J_{t}$ in the state vector allows the estimator to react very rapidly to the change in aerodynamic parameters once the icing hypothesis becomes dominant among the particle set. The transition following the onset of icing is clearly visible, with the estimates converging toward the true values almost immediately after ice detection. Post-detection, the estimator remains stable and consistent, indicating that particle degeneracy is mitigated through resampling.

Figure 6b shows the evolution of the icing probability computed from the empirical ratio in (28). The probability exhibits a step-like increase once the icing hypothesis is accepted by a majority of particles, in contrast to the gradual response of the Gaussian kernel method. This abrupt rise reflects the discrete nature of the detection mechanism. The method enables faster detection but is sensitive to the selection of the jump probability $p_{c}$, which must be properly tuned to balance responsiveness and robustness.

**Figure 7:**
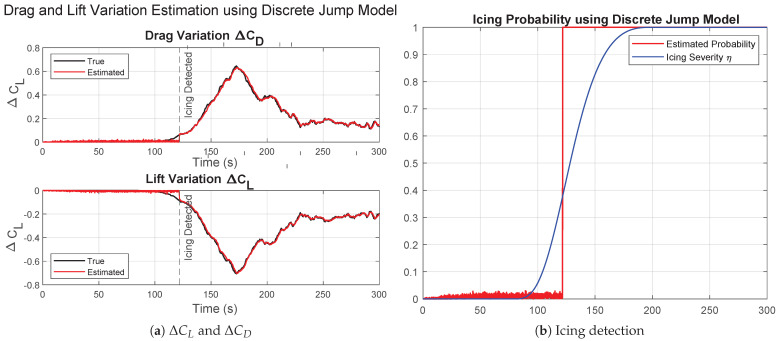
Results for severe icing using the second approach, discrete jump model.

The results for severe icing using the discrete jump model are shown in Figure 7.

In this case, aerodynamic degradation occurs rapidly, leading to large variations in $\Delta C_{L}$ and $\Delta C_{D}$. The estimates closely follow the true changes with minimal delay. The icing probability increases sharply to unity soon after $t_{occ}=80\;s$, as particles associated with the icing hypothesis acquire dominant weights following the resampling step. The results confirm that the discrete jump framework is well-suited for abrupt parameter transitions, while still preserving estimation stability due to the Gamma-distributed noise generation defined in (24) and (25).

### 8.3. Quantitative Performance Evaluation

Table 1 summarises the state estimation goodness of fit results in terms of mean square error (MSE), normalised mean square error (NMSE), and normalised root mean square error (NRMSE). These metrics are used to assess the accuracy of the proposed Gaussian kernel and discrete jump particle filters and for the EKF used for comparison under moderate and severe icing conditions. The MSE, NMSE, and NRMSE are computed using vector formulations, where the estimation error is expressed as the Euclidean norm of the error vector. The results tables represent a typical median result (in terms of MSE) from 50 Monte Carlo runs.

The two particle filtering approaches outperform the EKF in terms of estimation accuracy and infinity norm (maximum deviation) errors. The PF approaches yield comparable estimation performance. Under moderate icing, the PF with Gaussian kernel-based ice detection exhibits slightly lower MSE values, which can be attributed to the continuous probabilistic weighting that smooths out small fluctuations. The PF with a discrete jump model achieves very similar results, confirming that the addition of a discrete jump indicator does not degrade estimation accuracy. Under severe icing, both approaches converge toward similar error levels, as the dominant aerodynamic deviations overshadow noise-induced variations.

The corresponding detection times are summarised in Table 2.

In the moderate icing case, the discrete jump model detects icing earlier (approximately 123.9 s) compared to the Gaussian kernel approach (approximately 162.8 s) and the EKF. This improvement in detection latency arises from the use of guided noise and probabilistic jumps that allow for rapid hypothesis switching once sufficient evidence is accumulated. Under severe icing, all approaches achieve similar detection times (differences below 2 s) because the strong aerodynamic deviations facilitate fast identification, regardless of the detection strategy, with uniform noise. The results under mixed Gaussian noise are not listed for brevity, but the main observation in this case was that while the two particle filters performed similarly to the case of uniform noise for the icing detection under both severe and moderate icing, the EKF typically detected icing before its onset (false alarm), which is attributed to the cumulative effects of its noisy characteristic with the heavier tail of mixed Gaussian noise. Indeed, the large high-frequency oscillations in the detection probability make the EKF more prone to false alarms. The evaluation and minimisation of detection error probability remains an area for further investigation using the proposed ice detection and estimation algorithms.

The comparative analysis highlights the advantages of the particle filtering approach compared to the EKF as well as the trade-offs to be considered for choosing the right PF approach from the two PF detection paradigms. The Gaussian kernel approach offers a continuous probabilistic indication of icing, suitable for gradual accretion with low false alarm rates but a slower response. The discrete jump model explicitly handles abrupt regime changes, providing faster detection with a limited increase in the sensitivity to noise by modelling noise as gamma distribution with parameters ($p_{c}$, $k_{0}$, $\theta_{0}$). Those parameters are adjusted depending on the abrupt or incipient nature of the icing event. Adaptive tuning of these parameters is a natural future research direction.

Both methods demonstrate the ability to handle nonlinear, uncertain, and non-Gaussian conditions, confirming the suitability of particle filtering for joint icing detection and aerodynamic state and parameter estimation in UAVs. The selection between the proposed approaches should depend on mission requirements, including detection speed and reliability, as well as the phase of flight, icing type (e.g., small ice droplets, supercooled large droplets), and severity.

## 9. Conclusions

This paper presented two particle filtering-based approaches for in-flight icing detection and aerodynamic state estimation in fixed-wing unmanned aerial vehicles. Both methods address the limitations of conventional model-based or sensor-dependent schemes by relying solely on standard onboard measurements and incorporating aerodynamic degradation directly into the state estimation process. The first approach employs a Gaussian kernel function to compute the probability of icing based on deviations in the estimated lift and drag coefficients. It provides a gradual probabilistic response suited to slowly evolving icing conditions. The second approach introduces a discrete jump model within the particle filter framework, using a binary indicator and Gamma-distributed process noise to capture abrupt aerodynamic changes. This formulation enables faster detection and maintained stable estimates following icing onset.

Simulation results for nonlinear UAV dynamics under moderate and severe icing conditions demonstrate that both methods outperform the EKF with a more accurate estimation of aerodynamic parameter variations and of the icing probability, also allowing for more reliable icing detection. This comparison was validated under moderate to severe deviations from the normal distribution in both sensor noise and model uncertainty. The Gaussian kernel approach yields smoother probabilistic evolution, while the discrete jump model enhances detection responsiveness. The EKF produces a much noisier estimate of icing probability, which implicitly suggests a higher risk of false alarms and missed detections compared to the particle filtering approaches.

Future work will initially focus on validating the performance of the proposed algorithms and evaluating the detection error probability through postprocessing of real flight data, as a step towards validation on embedded UAV platforms. Additional research should investigate the adaptive tuning of filter parameters to robustly handle dynamic variations in the measurement and process models, as well as integration with energy-efficient de-icing control strategies for autonomous operation.

## Figures and Tables

**Table 1:** Performance comparison of the particle filtering and EKF approaches for moderate and severe icing.

Method	Case	MSE	NMSE	NRMSE
PF with Gaussian kernel	Moderate	0.0234	0.4058	0.5997
	Severe	0.0168	0.3088	0.2326
PF with a discrete jump	Moderate	0.0253	0.4084	0.5882
	Severe	0.0228	0.0298	0.2365
EKF	Moderate	0.0442	0.4482	0.7835
	Severe	0.0245	0.0651	0.3094

**Table 2:** Summary of detection time using both methods, occurrence time, and final time in both moderate and severe icing.

Condition	*t_occ_*	*t_occ_* + *T_cld_*	PF-Gaussian Kernel	PF-Discrete Jump	EKF
Moderate Icing	80	280	162.8	123.9	159.4
Severe Icing	80	200	121.8	121.4	122.8

## Data Availability

The data presented in this study are available upon approved request from the first author, due to institutional ethical research and data management processes.
